# Effect of mouthrinses on color stability of monolithic zirconia and feldspathic ceramic: an in vitro study

**DOI:** 10.1186/s12903-017-0419-9

**Published:** 2017-11-07

**Authors:** Reza Derafshi, Hooman Khorshidi, Mohamadhasan Kalantari, Ilyad Ghaffarlou

**Affiliations:** 1 Biomaterials Research Center, School of Dentistry, Shiraz University of Medical Sciences, Shiraz, Iran; 2 Department of Periodontics, School of Dentistry, Shiraz University of Medical Sciences, Shiraz, Iran; 3 Department of Prosthodontics, School of Dentistry, Shiraz University of Medical Sciences, Shiraz, Iran

**Keywords:** Color, Chlorhexidine, Ceramic, Listerine, Mouthrinse

## Abstract

**Background:**

Patients susceptible to periodontal disease and dental caries, including those who undergo fixed prosthodontic treatments use chemical plaque control agents. However, these mouthrinses may result in adverse effects such as discoloration of the restorative materials. The aim of this study was to compare the color stability of monolithic zirconia and feldspathic porcelain after immersion in two different mouthrinses: 0.2% Chlorhexidine digluconate (CHX), or Listerine®. Color change was evaluated by color spectrophotometer and according to the Commission Internationale de l’Eclairage (CIELab) system.

**Methods:**

We prepared 72 disc-shaped porcelains (*n* = 36) as follows: Group A consisted of dental direkt cube X2 discs (49% translucency) as the monolithic zirconia and group B consisted of VITA VMK 95 as a feldspathic porcelain. Groups A and B were divided into three subgroups (*n* = 12 per group). Each subgroup was immersed in one of the following three solutions: distilled water (control), CHX, or Listerine® for 2 min, once per day. We recorded the samples’ baseline color values according to the CIELab system by using a color spectrophotometer operated by an experienced operator. Color measurements were subsequently obtained following 7 days of immersion, and after the samples were rinsed with distilled water and allowed to dry. We measured CIE L*, a*, and b*and calculated the color difference (ΔE*ab). All data were analyzed by the Mann-Whitney and Kruskal-Wallis tests.

**Results:**

Color changes occurred in the experimental groups. The ΔE*ab values were significantly greater in VMK 95 porcelain compared to cube X2 (both *p* < 0.001) following immersion in CHX and Listerine® mouthrinses. However no significant difference was founded when distilled water was used (*p* = 0.630). For the two materials, the ΔE values were highest in CHX, followed by the Listerine® and distilled water.

**Conclusion:**

Both monolithic zirconia and feldspathic porcelain were susceptible to color changes following immersion in CHX and Listerine® mouthrinses.

## Background

The demands for esthetic restorations among patients encourage researchers to develop more esthetic metal-free ceramic restorations. Characteristics of traditional ceramics include biocompatibility and high esthetics; however, brittleness and low tensile strength of the materials have led to the development of an alternative choice, zirconia crown, which has high mechanical and biological properties [1, 2]. Although there are three types of zirconia, Yttrium stabilized tetragonal zirconia polycrystalline (Y-TZP) is most frequently used due to its high esthetics, excellent biocompatibility, and increased resistance to fractures [3–5]. Y-TZP zirconia has yettria (Y_2_O_3_) or ceria (CeO_2_) and a white-opaque appearance [1]. Therefore it should be covered with a more translucent ceramic layer [3, 4, 6]. Recently, improvements in translucency and various coloring technologies have enabled researchers to match the natural tooth color. Zirconia ceramic has been developed to a monolithic design for dental applications [1, 7].

Although tooth color restorative materials provide esthetics, discoloration of these materials present challenges for dentists. According to research, the color of the restorative materials may be influenced by plaque accumulation, stains from solutions, surface roughness, and chemical degradation, all of which may result from consumption of different beverages (tea, coffee) or the use of mouthrinses [8, 9].

Oral hygiene is an important factor for preventing dental caries and/or treatment of gingivitis. Oral hygiene can be maintained by routine mechanical dental plaque removal in addition to the use of chemical therapeutic agents. Dentists prescribe chemical plaque control agents for patients susceptible to periodontal disease and dental caries such as those who undergo fixed prosthodontic treatments. Chlorhexidine digluconate (CHX) is a widely recognized antibacterial agent that reduces periodontal disease and dental caries. CHX is prescribed as a gel, spray, or mouthrinse. Adverse effects that follow CHX administration include staining of the enamel or restorative materials, calculus formation, and temporary unpleasant taste [10, 11]. According to research, there is discoloration of restorative materials following their immersion in CHX mouthrinse [12, 13]. Listerine® is a mouth rinse used as an anti-plaque agent to treat gingivitis. Initially, this mouth rinse contained four essential oils - peppermint, eucalyptus, thyme, and wintergreen, which were later replaced by menthol, eucalyptol, thymol, and methyl salicylate. Listerine® includes 24%–27% ethanol as the vehicle to maintain the phenolic component solvents [14, 15]. However, composite resin discoloration has been reported following the use of Listerine® mouthrinse [15].Other common mouthrinses contain fluoride ingredients. Researchers have also reported ceramic discoloration and surface roughness after the use of fluoride products [8, 16].

Initial attempts to organize tooth color matching were performed by Clark based on the Munsell color scale [17] after which the Commission International de l’Eclairage introduced the first standard for color matching [18]. Both the systems showed some irregular distribution between color calculation and color perception. From 1976 to 78, CIE introduced a new scientific system - CIELab*that reported color by number and calculated color differences. The CIELab system is a uniform color scale where L represents lightness and b describes chromatic characteristics. In this system, “a” is the green/red coordinate and “b” represents the blue/yellow coordinate (−a = green, +a = red, −b = blue, and +b = yellow). The color differences are reported by delta values - ΔL*, Δa*, and Δb* compared with standard conditions. The total color differences (ΔE*ab) indicate differences between L*, a*, and b* of the sample and standard. These differences are calculated according to the following formula: [19].

$$ {\Delta \mathrm{E}}^{\ast}\mathrm{ab}=\left[\left({\Delta \mathrm{L}}^{\ast}\right)2+\left({\Delta \mathrm{a}}^{\ast}\right)2+\left({\Delta \mathrm{b}}^{\ast}\right)2\right]1/2 $$

In the point of clinical assessment, if ΔE*ab is ≤ 3.3, the color changes in the restoration are acceptable [20, 21]. In 2004, CIE developed Δab to a new formula ΔE_00_ to provide better human perceptibility and acceptability of color differences between tooth colors [22]. Perceptibility is defined as the difference between colors detectable by the human eye whereas acceptability refers to tolerable differences between colors. Another formula, CIEDE2000, provides a better assessment of color differences among dental ceramics [23]. However, the majority of research articles have used ΔEab for detections in color change due to the complexity of the ΔE_00_ formula [20].

There are conflicting opinions regarding thresholds for perceptibility and acceptability of different dental materials. Douglas et al. have reported the lower thresholds for perceptibility (mean ΔE = 0.4 unit, ΔE = 2.6 unit) than acceptability (1.7 unit ΔE, 5.5 unit ΔE) for metal ceramic crowns and denture teeth, respectively [24, 25]. However, Lindsey did not find any significant difference between them [26].

Ghinea et al. [23] used ΔEab and the new ΔE_00_ formula for color calculation and reported a significant difference between perceptibility and acceptability thresholds for dental ceramics.

Following the scientific approach to tooth color matching, some devices were introduced to color measurement. A colorimeter is a simple, inexpensive instrument to measure color based on three axes or stimuli such as the human eye. The spectrophotometer is a device developed to measure color by reflection or transmission of an observed object. The spectrophotometer is a commonly used accurate instrument that records color changes in restorative materials. User friendly and electronic instruments were subsequently introduced such as CCDs and fiber optics [20].

We have not found any data that pertained to the use of mouthrinses with new monolithic zirconia. Hence, this in vitro study aimed to compare the effects of two mouthrinses, CHX and Listerine®, on feldspathic porcelain and monolithic zirconia.

## Methods

**Fig. 1:**
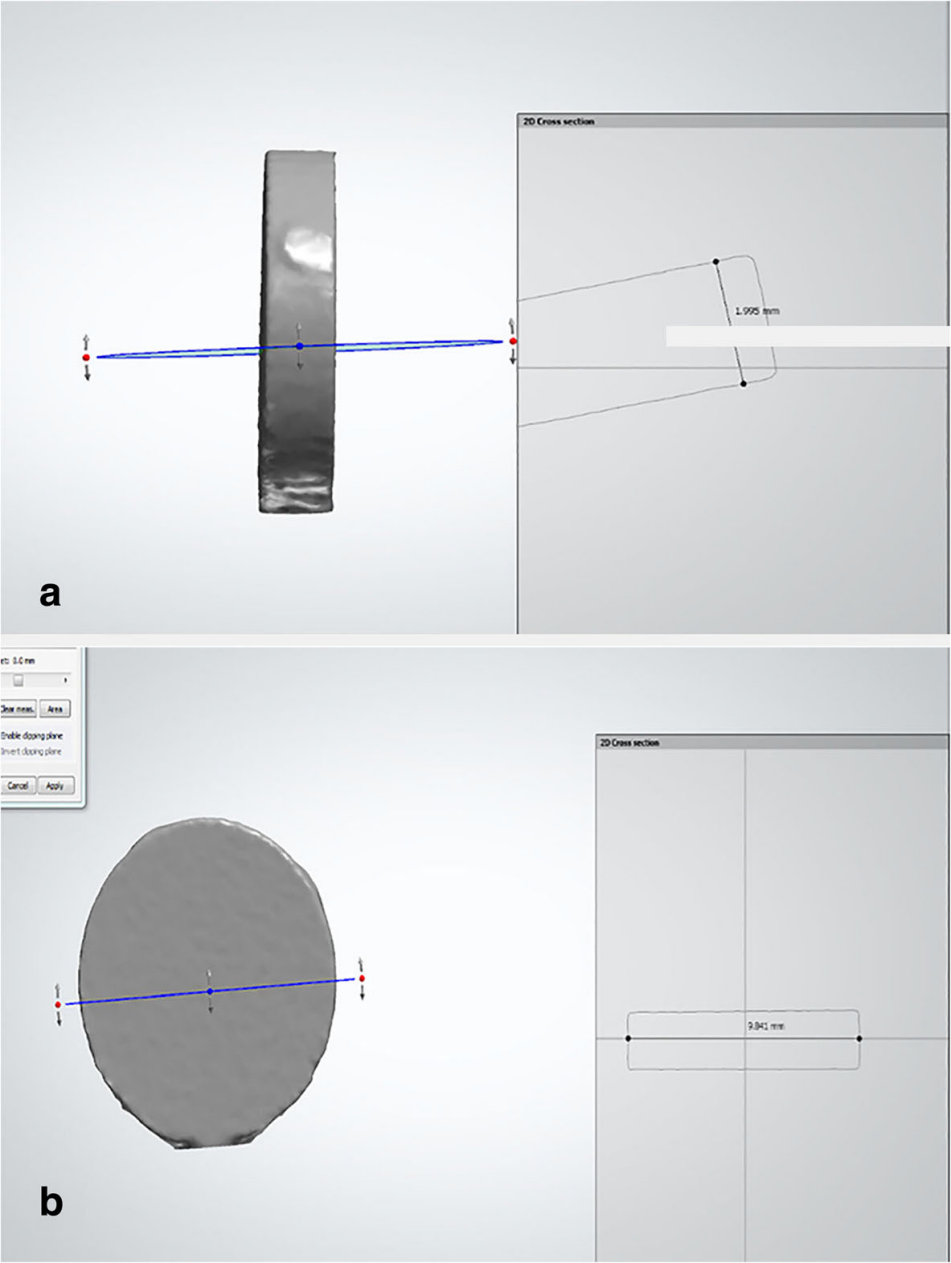
(**a**, **b**): Illustrations of scanned disc by CAD/CAM System

We prepared 72 ceramic discs as follows: Group A consisted of specimen discs made of dental direkt cube X2 (Spenge, Germany) with 49% translucency as the monolithic zirconia. The initial 36 wax inlay discs had a 10 mm diameter and 2 mm thickness. These discs were prepared and scanned by a Cercon Eye unit (Dentsply Ceramco; Burlington, NJ, USA). Next, the cube X2 pre-sintered zirconia blank (Dental Direkt GmbH, Spenge, Germany) were mounted on a Cercon Brain unit (computer-aided design and computer-aided manufacturing; CAD/CAM system) and milled with tungsten carbide burs with diameters of 2.8 mm and 1.0 mm (Fig. 1). The milled ceramic discs were fired at 1450 °C for 6 h and trimmed. A standardized polish was performed where the discs were sequentially machine-polished with wet 320, 400, 600, 800, 1000, and 1200 grades of silicon carbide papers to 0.5 ± 0.05 mm thickness, followed by cleansing.

The group B sample discs were prepared from VITA VMK 95 (Vita Zahnfabrik, Bad Säckingen, Waldshut, Germany) as the feldspathic porcelain. For this purpose, we prepared 36 wax inlay discs that were 10 mm in diameter with a 0.7 mm thickness. The discs were sprued and casted by phosphate bonded investment material. Then, each disc surface was sandblasted with 100 μm aluminumoxide abrasion particles to remove any remaining investment. Carborundum discs and metal trimmers were used to finish the metal discs to achieve a uniform thickness. Dimensions of the prepared specimen discs were approximately10 mm in diameter and 0.5 mm thickness. Next, the samples were covered by VITA porcelain (VITAVMK 95) according to the manufacturer’s instructions. Two coats of paste opaque were applied and fired. We prepared the dentine layer by mixing powder and liquid, which were applied over the samples by a metal jig. The enamel layer was then applied with a similar technique used for the dentine layer and the discs were fired. The discs were finished with a diamond bur to achieve a uniform thickness of 2 mm and subsequently glazed. We immersed the discs in 75% ethanol in an ultrasonic bath for 10 min for a final cleansing, after which they were dried.

Of note, we selected shade A2 from the VITA Lumin shade guide (Vita ZahnFabrik, Bad Säckingen, Waldshut, Germany) as the standardized initial color for all specimens in both groups. The firing procedure in the study was performed according to the manufacturer’s instructions.

Initially, we measured the color values (L*, a*, b*) for the specimens in all of the groups. This measurement was considered the baseline color. The measurement was performed by a calibrated reflectance spectrophotometer (SpectroShade, Handy Dental Type, MHT, Arbizzano di Negar, Verona, Italy) against a white background. The device was kept perpendicular to the surfaces to guarantee similar conditions for all of the samples. Before each measurement, we calibrated the spectrophotometer according to the manufacturer’s recommendations. All measurements were performed under a D65 light source **(**GL OptiLight LED 127 CLC, USA).

**Fig. 2:**
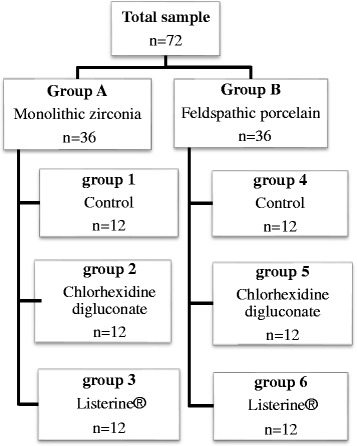
Flowchart showing the methods and materials used in this study

We stored each of the prepared discs in distilled water for 24 h at 37 °C. Groups A and B were randomly divided into three subgroups each (*n* = 12 per group) according to the solutions used and then the baseline color measured. The sample size was determined to *n* = 12 in each subgroup according to comparison of mean ΔE between Listerine®/ Chlorhexidine with distilled water [27]; α = 0.05 and power (1-β) = %80.We treated the prepared discs as follows (Fig. 2):

In the distilled water (control, groups 1 and 4) had no intervention. The samples were immersed in10 ml distilled water for 2 min once per day. The water was changed every day for 7 days.

In the CHX groups (groups 2 and 5),each specimen was immersed in 10 ml of 0.2% CHX mouthrinse (Shahrdarou Co., Tehran, Iran, non-alcohol base mouthrinse) at a pH of 5.1 and 37 °C for 2 min, once per day [28]. The solution was replaced every day for 7 days.

In the Listerine® (groups 3 and 6) subgroups, each disc was immersed in 10 ml Listerine® mouthrinse (Listerine® Tooth Defense Antic, Johnson & Johnson, Italy, alcohol base mouthrinse) that had a pH of 4.2 and 37 °C for 2 min, once per day. The solution was replaced every day for 7 days.

We stored each sample in artificial saliva (Caphosol® Cytogen Corp. Princeton NJ, USA) in separate closed containers. The saliva was replaced daily with new saliva. Color measurements were performed 7 days later, after which each disc was washed with distilled water and dried with paper.

In group B (VITA VMK 95), one side of each disc was covered with metal. The other side was exposed to the solutions. Therefore, the color measurement was accurate. However in group A (dental direkt cube X2), both sides of the disks were in contact with the solutions. The procedure might alter the color measurement due to created colored background. We increased the accuracy of the color measurement by polishing one side with 600, 800, and 1200 grit discs for 10 s after which we recorded the color values from the other side.

### Statistical analysis

Data were described using median mean and standard deviation (SD) measures. Kruskal-Wallis H test and Mann-Whitney U test were used for comparisons of different solutions and materials. SPSS version 18.0 (SPSS Inc., Chicago IL, USA) was used for statistical analysis. A *p* < 0.05 was considered statistically significant.

## Results

Table 1 shows the mean and standard deviation of the baseline and final L, a, and b values. After we immersed the two materials in CHX, we observed a significant difference in Δa and Δb between dental direkt cube X2 and VMK 95 porcelain (both *p* < 0.001). There was no significant difference in ΔL values (*p* = 0.378).

**Table 1:** Median (mean ± SD) of baseline and final change in a, b and L indices according to the material and solutions

Mouth-rinse	ΔL		Δa		ΔL	
	Cube X2	VMK 95	Cube X2	VMK 95	Cube X2	VMK 95
CHX	−0.20^A,a^ (−0.23 ± 0.87)	−0.20^A,a^ (0.18 ± 0.99)	0.70^A,a^ (0.68 ± 0.09)	1.00^A,b^ (1.00 ± 0.06)	−0.40^A,a^ (−0.38 ± 0.12)	−0.50^A,b^ (0.53 ± 0.10)
Listerine®	−0.20^AB,a^ (−0.15 ± 0.14)	−0.20^A,a^ (−0.23 ± 0.9)	0.50^A,a^ (0.53 ± 0.20)	0.80^B,a^ (0.73 ± 0.27)	0.30^B,a^ (0.17 ± 0.27)	−0.20^B,a^ (−0.23 ± 0.89)
Distilled water	0.00^B,a^ (−0.67 ± 0.10)	0.00^B,a^ (−0.14 ± 0.12)	0.00^B,a^ (−0.33 ± 0.15)	0.0^C,a^ (−0.06 ± 0.14)	0.00^B,a^ (0.08 ± 0.37)	−0.10^C,a^ (−0.6 ± 0.11)

In each column, different superscript capital letters indicate significant differences between the materials. In each row, different superscript lower-case letters indicate significant differences between the solutions (Tukey HSD test, *p* < 0.001)

After we immersed the two materials in Listerine®, there was a significant difference observed in the Δa values (*p* = 0.001). There was no significant difference in Δb (*p* = 0.410) and ΔL (*p* = 0.319).There was no significant difference between Δa, Δb, and ΔL following immersion in distilled water (control).

**Fig. 3:**
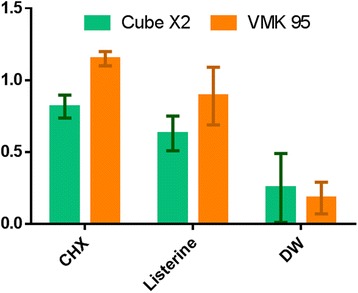
Mean ± SD of ΔE*ab values for each material after immersion in solutions

Table 2 and Fig. 3 show the descriptive statistics of the color difference values (ΔE*ab) of the baseline and final measurements of the two groups immersed in different solutions. There was a significant difference between the ΔE values of the two materials following immersion in CHX or Listerine® (both *p* < 0.001). In the CHX and Listerine® mouthrinses, we observed a significantly greater mean ΔE in VMK 59 porcelain compared to Cube X2 (both *p* < 0.001). However, there was no significant difference between the materials after immersion in distilled water (*p* = 0.630).

**Table 2:** Median (mean ± SD) of baseline and final color change (ΔE) according to the materials and solutions

Materials	Chlorhexidine	Listerine®	Distilled Water	*P* value
Cube X2	0.82^A,a^ (0.82 ± 0.08)	0.64 ^A,b^ (0.63 ± 0.12)	0.14 ^A,c^ (0.25 ± 0.33)	< 0.001
VMK 95	1.16 ^B,a^ (1.15 ± 0.05)	0.91 ^B,b^ (0.90 ± 0.20)	0.22 ^A.c^ (0.18 ± 0.11)	< 0.001
*P* value	< 0.001	< 0.001	0.630	

In each column, different superscript capital letters indicate significant differences between the materials. In each row, different superscript lower-case letters indicate significant differences between the solutions (Tukey HSD test, *p* < 0.001)

There was a significant difference in the mean ΔE between the three solutions (all *p* < 0.001). The mean ΔE values were highest in CHX, followed by Listerine®, and distilled water.

## Discussion

The current study assessed the effect of two mouthrinses on color stability of two different ceramic materials. For a number of years, the tooth shade guide was used to determine tooth color. Although inaccurate and subjective, this method was easy to use. Later, scientific methods have been introduced that overcame deficiencies with the visual guide. A spectrophotometer is an instrument that detects color changes. The spectrophotometry data can be translated into quantitative values. The advantages of the spectrophotometer include “accuracy, ability to analyze the principal components of a series of spectra, and the ability to convert data to various color measuring systems”. However the instrument is expensive, difficult to use, and mostly used by researchers [20]. Our results revealed that mouthrinses increased the staining ability of the two main groups. A number of researchers have stated that the ability of mouthrinse solutions to change the color of restorative materials depends on the type of restorative materials and the capability of resin matrixes to absorb water, in addition to the type of filler and filler content in resin composite restorations [29–31]. One study reported that the stain susceptibility of ceramic and CAD/CAM resin composites were less than methacrylate based direct composite [32]. According to Arocha et al., the color stain ability of two indirect CAD/CAM processed composites was more than two conventionally laboratory-processed [33]. According to the results of the current study, a significant difference existed between groups A and B following immersion in CHX or Listerine® mouthrinses. This finding agreed with a previous study that examined the color stability of resin composites [19, 34]. Festuccia et al. reported that greater discolorations of two resin composites occurred with Listerine® compared to Plax alcohol-free and Periogard CHX [35].

In the current study, we used two types of materials. Feldspathic porcelain contains quartz (SiO2), potassium aluminum silicate (orthoclase), and sodium aluminum silicate albite. The metal-ceramic restorations reconstruct in two phases- a lower melting point glass phase and a high-expansion phase that contained crystals - Leucite (KAlSi:O6). This combination provided a lower porcelain melting point compared to the metal melting point as well as a close thermal expansion coefficient of porcelain and metal [36].

We also used zirconia ceramic. Zirconia (ZrO2) was introduced as a core material with high mechanical properties that include high flexural strength (900–1200 MPa) and fracture toughness (9–10 MPa¢m1/2) [37]. We covered the ceramic with a veneer layer to increase the esthetic property. However, the veneer layer compared to the zirconia core had a number of disadvantages that included clinical failure due to chipping of this layer, differences in the coefficient of thermal expansion of the materials [38, 39], low fracture toughness, and flexural strength of the veneer ceramic [39]. Therefore, methods have been developed to overcome problems with the veneer layer. These methods include sintering a high-strength CAD/CAM fabricated veneering porcelain cap onto a zirconia coping [40]. The full contour monolithic zirconia has certain advantages to previous zirconia restorations with increased use by clinician. Monolithic zirconia provides high esthetic results as well as high fracture resistance even at a minimum thickness. There is no disadvantage to the use of a polished zirconia antagonist to the enamel and feldspathic porcelain [2–4]. In the present study the materials showed different mean ΔE. Generally, in full ceramic crowns, light transmission and translucency depend on the “crystal content, its chemical nature, particle size, and the thickness of the core” [41]. In the current study, we used the same thickness of materials in each group. The high physical properties in the zirconia group, may influence relative color stability of the material compared to feldspathic porcelain. However both materials included grain and small particles which might reduce surface roughness and susceptibility to discoloration. In addition, the crystalline structure of zirconia might decrease color changes compared to the feldspathic groups which contained more glass matrix [20].

Discoloration of restorative materials is determined by visual inspection and instrumental analysis. The latter is more accurate due to removal of subjective errors [42]. In the present study, we have measured the color changes via a CIELab system. In this system, ΔE < 1 is clinically acceptable and not perceptible to most subjects who have normal color vision. However, ΔE ≥ 3.3 indicates clinically perceptible color change and requires replacement of the restoration [20, 21].We have calculated an ΔE < 3.3 for both materials in all solutions, which was not visually perceptible. Baig et al. reported lower ΔE values for nanofilled resin composites immersed in Listerine® compared to non-alcohol CHX, which supported with the current study findings [34]. However, the ΔE values in their study were greater than the current study. In contrast Soygun et al. reported mouthrinses with higher alcohol content lead to increase color changes in bioceramic materials [27]. The discrepancies between different reports might be attributed to the types of materials (resin composite vs. ceramic) exposed to the mouthrinse solutions and duration of contact with the solutions and surface texture following different surface treatments [42, 43]. The measured color depends on both the actual colors of the surface and the lighting conditions. In the present study we have used standard lighting against a white background [44].

Mouthrinses are commercially available in two forms– alcohol-free or alcohol-based in which the alcohol mainly acts as the solvent [16]. In the present study we did not observe any correlation between pH and alcohol-based solutions in terms of discoloration. This finding agreed with previous studies that examined discolorations of resin composite resin [29, 34].

One of the potential limitations of the current study was that this in vitro study did not reflect clinical situations. In the current study we used artificial saliva to simulate an in vivo study. However, salivary pellicle and consumption of different foods and beverages might influence the color change susceptibility [45]. Further researches should compare the color stability of ceramics with different types of mouthrinses under clinical conditions.

## Conclusion

The present study showed that both monolithic zirconia and feldspathic porcelain underwent color changes after immersion in CHX and Listerine® mouthrinses. However, these changes were not visually perceptible. The color changes of both materials were within clinically acceptable ranges. Therefore, patients should feel confident using the mouthrinses, especially with zirconia crowns and Listerine®.

## Abbreviations

CAD/CAM: Computer-aided design and computer-aided manufacturing
CHX: Chlorhexidine digluconate
CIELab: Commission Internationale de l’Eclairage
Y-TZP: Zirconia polycrystalline
